# Alterations in the Rate of Limb Movement Using a Lower Body Positive Pressure Treadmill Do Not Influence Respiratory Rate or Phase III Ventilation

**DOI:** 10.1155/2015/618291

**Published:** 2015-01-14

**Authors:** Michael J. Buono, Marissa Burnsed-Torres, Bethany Hess, Kristine Lopez, Catherine Ortiz, Ariel Girodo, Karen Lolli, Brett Bloom, David Bailey, Fred W. Kolkhorst

**Affiliations:** Department of Biology, School of Exercise and Nutritional Sciences, San Diego State University, San Diego, CA 92182-7251, USA

## Abstract

The purpose of this study was to determine the effect of alterations in rate of limb movement on Phase III ventilation during exercise, independent of metabolic rate, gait style, and treadmill incline. Subjects completed five submaximal exercise bouts on a lower body positive pressure treadmill (AlterG P 200). The percent body weight for the five exercise bouts was 100, 87, 75, 63, and 50% and each was matched for carbon dioxide production (*V*_CO_2__). Naturally, to match the *V*_CO_2__ while reducing the body weight up to 50% of normal required a significant increase in the treadmill speed from 3.0 ± 0.1 to 4.1 ± 0.2 mph, which resulted in a significant (*P* < 0.05) increase in the mean step frequency (steps per minute) from 118 ± 10 at 3 mph (i.e., 100% of body weight) to 133 ± 6 at 4.1 mph (i.e., 50% of body weight). The most important finding was that significant increases in step frequency did not significantly alter minute ventilation or respiratory rate. Such results do not support an important role for the rate of limb movement in Phase III ventilation during submaximal exercise, when metabolic rate, gait style, and treadmill incline are controlled.

## 1. Introduction

Even after a century of investigation the control mechanisms responsible for the hyperpnea seen during steady state exercise (i.e., Phase III) are still under debate. One area that is particularly controversial is the role that limb movement has in exercise hyperpnea [1, 2]. A common experimental design used in the past to investigate the topic has been to alter the rate of limb movement during inclined treadmill walking versus horizontal treadmill running, while matching the metabolic cost of the two gait styles [3–7].

Collectively a dichotomous situation exists in the literature with some of these studies supporting the importance of limb movement frequency in exercise hyperpnea [5, 7, 8] while others do not [3, 4, 6, 9]. One common potential concern with the methodological design used in these past investigations is that two different gait styles (i.e., inclined walking versus horizontal running) were used to alter the frequency of limb movement.

Three lines of reasoning support the contention that controlling gait style may be important when examining exercise hyperpnea. First, studies have shown that both gait style and treadmill incline significantly affect the pattern of muscle recruitment, Achilles tendon stretch, and muscle strain in the lower limbs during exercise [10–12], all of which may alter exercise hyperpnea. Second, it is known that subjects rate uphill walking as being more difficult when compared to horizontal running at similar metabolic rates and that “perception of effort” during exercise can clearly alter exercise hyperpnea [13, 14]. Lastly, it has been suggested that inclined walking alters the vestibular-originated drive to breath compared to horizontal running due to differences in head motion between the two gait styles [1].

In an attempt to reduce the potential problems associated with past studies that have used two different gait styles, we have incorporated the recently developed lower body positive pressure treadmill [15, 16]. Specifically, the research design used in the current study allowed us to reduce the body weight of walking subjects while simultaneously increasing the speed, thus matching the metabolic cost of each workload. For example, walking at 3 mph at 100% body weight resulted in the same oxygen uptake and carbon dioxide production as walking at 4.1 mph at 50% of the body weight. Therefore, in the current study we could independently alter the stepping rate and thus the rate of limb movement while allowing the subjects to only use one gait style. Such a methodological design alleviates some of the potential concerns associated with past studies. It was hypothesized that the rate of limb movement would not significantly affect Phase III ventilation during submaximal exercise, when metabolic rate, gait style, and treadmill incline are controlled.

## 2. Methods

### 2.1. Subjects

The subjects for this study were 10 healthy volunteers (4 males and 6 females). The group had mean (±SD) age, height, and weight of 31.3 ± 14.2 y, 169.1 ± 14.0 cm, and 67.3 ± 16.8 kg, respectively. The study was approved by the San Diego State University IRB and signed informed consent was obtained.

### 2.2. Experimental Procedures

Each subject completed five submaximal exercise bouts on a lower body positive pressure treadmill (AlterG P 200). This device consists of a treadmill enclosed within a waist-high plastic chamber that has clear sides for viewing the subject in contact with the treadmill belt. A seal is achieved between the subject and the chamber through the use of a neoprene skirt fitted around the subject's waist that fastens over the opening of the plastic chamber. For unweighting, the pressure inside the chamber was elevated above the external, ambient pressure using an air compressor, with body weight being measured by a force transducer under the treadmill belt. The pressure differential caused an axial buoyant force that lifts the subject off the treadmill belt until the desired percent reduction in body weight is achieved. The percent body weight for the five exercise bouts was 100, 87, 75, 63, and 50% and the order was randomly assigned to each subject. Each exercise bout was between 5 and 8 min in duration as the subject walked at a 5% incline.

Oxygen uptake (*V*_O_2__), carbon dioxide production (*V*_CO_2__), minute ventilation (*V*_E_), and respiratory rate were measured every minute during the exercise bouts via a low resistance one-way valve attached to a calibrated metabolic cart (TrueOne, ParvoMedics, Sandy, UT) which used a heated pneumotach (Hans Rudolph, Kansas City, MO) to measure expired ventilation. The pneumotach was calibrated before testing using a 3 L syringe. The treadmill speed was adjusted for each subject to match the *V*_CO_2__ during the five exercise bouts. Two consecutive *V*_CO_2__ readings within 0.1 L/min were considered as evidence that steady state values were obtained during each exercise bout, and the ventilatory and metabolic data collected for those two minutes were averaged for the recorded value. Step frequencies were counted by two investigators using a stopwatch during each exercise bout and were averaged. Repeated measures ANOVA and Tukey's post hoc tests were used to compare the data obtained during the 5 different body weight conditions. Significance was set at the *P* < 0.05 level.

## 3. Results

The mean (±SD) metabolic and ventilatory data collected for the 5 exercise bouts are presented in Table 1. As can be seen, we were able to match the metabolic rate as evidenced by the fact that the *V*_O_2__ and *V*_CO_2__ were essentially identical and not significantly different for the five exercise trials. Specifically, the mean *V*_CO_2__ ranged between 1.04 and 1.05 L/min for all five conditions. To match the *V*_CO_2__ while reducing the body weight up to 50% of normal required a significant increase in the treadmill speed from 3.0 ± 0.1 to 4.1 ± 0.2 mph. The increased treadmill speed resulted in a significant increase in the mean step frequency from 118 ± 10 steps per min at 3 mph (i.e., 100% of body weight) to 133 ± 6 at 4.1 mph (i.e., 50% of body weight).

The major finding of the current study was that increased step frequency did not alter *V*_E_ or respiratory rate. Specifically, mean *V*_E_ ranged between 32.1 and 33.4 L/min for the five different body weight conditions. Similarly, the mean respiratory rate was not significantly different for the five conditions, ranging between 25 ± 4 and 27 ± 6 breaths per minute for all five trials.

## 4. Discussion

It is clear that exercise hyperpnea likely requires multiple redundant control mechanisms, including various neural and humoral elements. The current study examined only one specific component, namely, the role of limb movement frequency in Phase III ventilation during rhythmic exercise [17]. The importance of limb movement frequency in ventilation during exercise is controversial and past investigators have used numerous different research designs to investigate the response.

One commonly used experimental design has been to alter the rate of limb movement during exercise while keeping the metabolic rate constant. To accomplish this, many investigators have compared the ventilatory responses obtained during inclined treadmill walking versus horizontal treadmill running, while matching the metabolic cost of the two gait styles. Collectively, some of these studies support the importance of the rate of limb movement in exercise hyperpnea [5, 7, 8, 18] while others do not [3, 6]. One potential concern with the methodological design used in those past investigations is that two different gait styles (i.e., inclined walking versus horizontal running) were used, which may alter the afferent feedback, independent of the rate of limb movement [4, 10–12].

In an attempt to reduce the potential problems associated with past studies that have used two different gait styles, we used a lower body positive pressure treadmill [15, 16]. Specifically, the research design incorporated in the current study allowed us to reduce the body weight of walking subjects while simultaneously increasing the speed, thus matching the metabolic rate of each workload. For example, walking at 3 mph at 100% body weight resulted in the same oxygen uptake and carbon dioxide production as walking at 4.1 mph at 50% of the body weight. Therefore, in the current study we could independently alter the stepping rate and thus the rate of limb movement while allowing the subjects to only use one gait style at the same treadmill incline. Such a methodological design alleviates some of the concerns associated with past studies.

The results of the current study do not support an important role for limb movement frequency during Phase III ventilation, as evidenced by the fact that neither mean *V*_E_ nor respiratory rate was significantly different during the five different body weight conditions. Such results agree with the findings of Berry et al. [3] and McMurray and Smith [6] but are in conflict with others [5, 7, 8, 18]. A logical question is the following: “Why is there such a discrepancy in the literature concerning the role of limb movement frequency in exercise hyperpnea?” We believe that the answer may lie in which phase (i.e., I, II, or III) of the ventilatory response is examined. For example, Berry et al. [3] were careful not to measure the ventilatory responses during inclined treadmill walking versus horizontal running until the subjects had reached the steady state Phase III level. This agrees with our methodology in that ventilatory parameters were not collected in the current study until the subject had two consecutive minutes of *V*_CO_2__ within 0.1 L/min of each other. Conversely, closer examination of past studies that have found limb movement frequency to significantly affect exercise hyperpnea reveals that their subjects were not in Phase III ventilation but rather most likely in Phase I or II. For example, Wells et al. [7] used sinusoidal changes in treadmill speed and grade to test the hypothesis that limb movement frequency is a significant determinant of exercise hyperpnea. Their findings suggested that changes in limb movement frequency did significantly influence exercise hyperpnea. However, during sinusoidal submaximal exercise the *V*_E_ responses are likely most comparable to Phase II, not Phase III, responses to a step change in exercise [2]. Interestingly, when they measured Phase III ventilatory responses during steady state inclined treadmill walking versus horizontal treadmill running at matched metabolic rates, they reported essentially identical mean *V*_E_ for the two gait styles (29.3 versus 30.4 L/min, resp.), which agrees with the current findings. Likewise, Hanson et al. [8] reported a significant 10–25% increase in *V*_E_ during horizontal treadmill running compared to inclined treadmill walking during high intensity exercise (≥75% maximal *V*_O_2__). However, it is unlikely that at such high exercise intensities Phase III ventilatory responses were obtained [19]. Incidentally, the *V*_E_ responses during the two gait styles were identical during lower intensity exercise (≤60% maximal *V*_O_2__), during which Phase III steady state ventilatory responses were most likely attained. The results of the current study, coupled with past findings, suggest that frequency of limb movement may be important during Phases I and II exercise hyperpnea; however, its role is attenuated if data is collected after the subject reaches Phase III [3, 7, 8].

In conclusion, this is the first study in the literature that has used a lower body positive pressure treadmill to alter the stepping frequency during treadmill walking while matching the *V*_CO_2__. The results of the current study suggest that frequency of limb movement is not important during Phase III exercise hyperpnea, when *V*_CO_2__, gait style, and treadmill incline are controlled.

## Figures and Tables

**Table 1:** Mean (±SD) physiological responses to reduced body weight exercise.

Body weight (%)	Walking speed (mph)	*V*_O_2__ (L/min)	*V*_CO_2__ (L/min)	Frequency (steps/min)	*V*_E_ (L/min)	Respiratory rate (breaths/min)
100%	3.0 ± 0.1	1.23 ± 0.36	1.04 ± 0.27	118 ± 10	33.0 ± 8.4	26 ± 4
87%	3.3 ± 0.2	1.20 ± 0.34	1.04 ± 0.25	124 ± 9^*^	32.1 ± 8.0	25 ± 4
75%	3.6 ± 0.2	1.23 ± 0.33	1.05 ± 0.24	129 ± 8^∗#^	32.9 ± 8.1	26 ± 5
63%	3.9 ± 0.1	1.20 ± 0.33	1.04 ± 0.26	133 ± 6^∗#⧫^	33.4 ± 8.4	27 ± 6
50%	4.1 ± 0.2	1.19 ± 0.33	1.05 ± 0.25	133 ± 6^∗#⧫^	33.1 ± 8.0	26 ± 5

^*^indicates that value is significantly (*P* < 0.05) different than the 100% BW value, ^#^indicates that value is significantly (*P* < 0.05) different than the 87% BW value, and ^⧫^indicates that value is significantly (*P* < 0.05) different than the 75% BW value.
